# Could Vegetation Index be Derive from Synthetic Aperture Radar? – The Linear Relationship between Interferometric Coherence and NDVI

**DOI:** 10.1038/s41598-020-63560-0

**Published:** 2020-04-21

**Authors:** Zechao Bai, Shibo Fang, Jian Gao, Yuan Zhang, Guowang Jin, Shuqing Wang, Yongchao Zhu, Jiaxin Xu

**Affiliations:** 1 State Key Laboratory of Severe Weather, Chinese Academy of Meteorological Sciences, Beijing, 100081 China; 2 Collaborative Innovation Center on Forecast and Evaluation of Meteorological Disasters, Nanjing University of Information Science & Technology, Nanjing, 210044 China; 3 North China University of Technology, Beijing, 100144 China; 4 Institute of Mordern Forestry, Xinjiang Academy of Forestry, Urumqi, 830063 China; 5 Zhengzhou Institute of Surveying and Mapping, Zhengzhou, 450052 China; 6 China University of Geosciences, Beijing, 100083 China

**Keywords:** Agroecology, Grassland ecology

## Abstract

Due to many factors in the physical properties of the ground surface, the corresponding interferometric coherence values change dynamically over time. Among these factors, the roles of the vegetation and its temporal variation have not yet been revealed so far. In this paper, synthetic aperture radar (Sentinel-1) data and optical remote sensing (Landsat TM) images over four whole seasons are employed to reveal the relationship between the interferometric coherence and the normalized difference vegetation index (NDVI) at five sites that have ground deformation due to mining in Henan province, China. The result showed: (1) As for the village area with few vegetation cover, the related coherence values are significantly higher than that in the farm land area with high densities of vegetation in the spring and summer, which indicates that the subsidence by mining in few vegetation cover area is easier to be monitored; (2) Linear regression coefficients (${{\bf{R}}}^{{\bf{2}}}$) between the interfereometric coherence values and the NDVI values is 0.62, which indicate the interferometric coherence values and the NDVI values change reversely in both farm land and village areas over the year. It suggests months between November and March with lower NDVI value are more suitable for deformation detecting. Therefore, the interfereometric coherence values can be used to detect the density of vegetation, while NDVI values can be reference for elucidating when the traditional differential interferometric synthetic aperture radar (DInSAR) could be effectively used.

## Introduction

DInSAR leverages the phase difference between two correlated synthetic aperture radar (SAR) images to accurately detect large scale surface displacements and is widely used for mine deformation monitoring^1–4^. However, as the technique suffers from a number of limitations, including spatial decorrelation, thermal noise, Doppler centroid shift, and temporal decorrelation, it is not appropriate in certain situations^5–7^. Some research address the limitations of traditional DInSAR disturbed by the agricultural activities, especially the high density of crop vegetation, in the test of the polarimetric InSAR (POLInSAR) technique for its ability to increase interferometric coherence^8,9^. Therefore, it is necessary to elucidate the deformation monitoring conditions under which the traditional DInSAR can be effectively used.

The coherence is also taken as the main parameter in target classification^10,11^, forest change detection^12–14^, and lake study^15,16^. The extent of temporal changes in the scatterers is a key factor affecting interferometric coherence^11,17^. In DInSAR deformation measurements, the interferometric coherence is used for selecting the stable scatterers to achieve better accuracy^5^. Compared with other scatterers, vegetation has a larger impact on SAR image coherence. In the seasons when vegetation is growing, the temporal decorrelation phenomena is particularly complicated^13,18^. SAR observations at different points during the crop-growing season are sensitive to growth stages, biomass development, plant height, soil moisture, and inundation frequency and duration^19,20^. The coherence degradation is particularly obvious in the condition of dense vegetation cover in summer. However, there are few studies focusing on interferometric coherence variations in the whole life cycle of vegetation growing and withering fluctuations as well as the relationship between interferometric coherence and vegetation density or coverage.

The objective of this work is to analyze the temporal changes of the coherence in SAR images and NDVI variation of optical remote sensing images within one year, to reveals the relationship between SAR image coherence and the vegetation density. The interferogram and interferometric coherence of a representative set of Sentinel-1A satellite images were computed via temporal evolution charts from June 2015 to May 2016, and multi-temporal NDVIs were obtained from a time series Landsat 8 satellite images from July 2015 to May 2016. The correlation analysis was performed to quantify the relationship between the multi-temporal interferometric coherence and NDVI throughout the year in mine deformation area.

## Results

### Study Area

**Figure 1:**
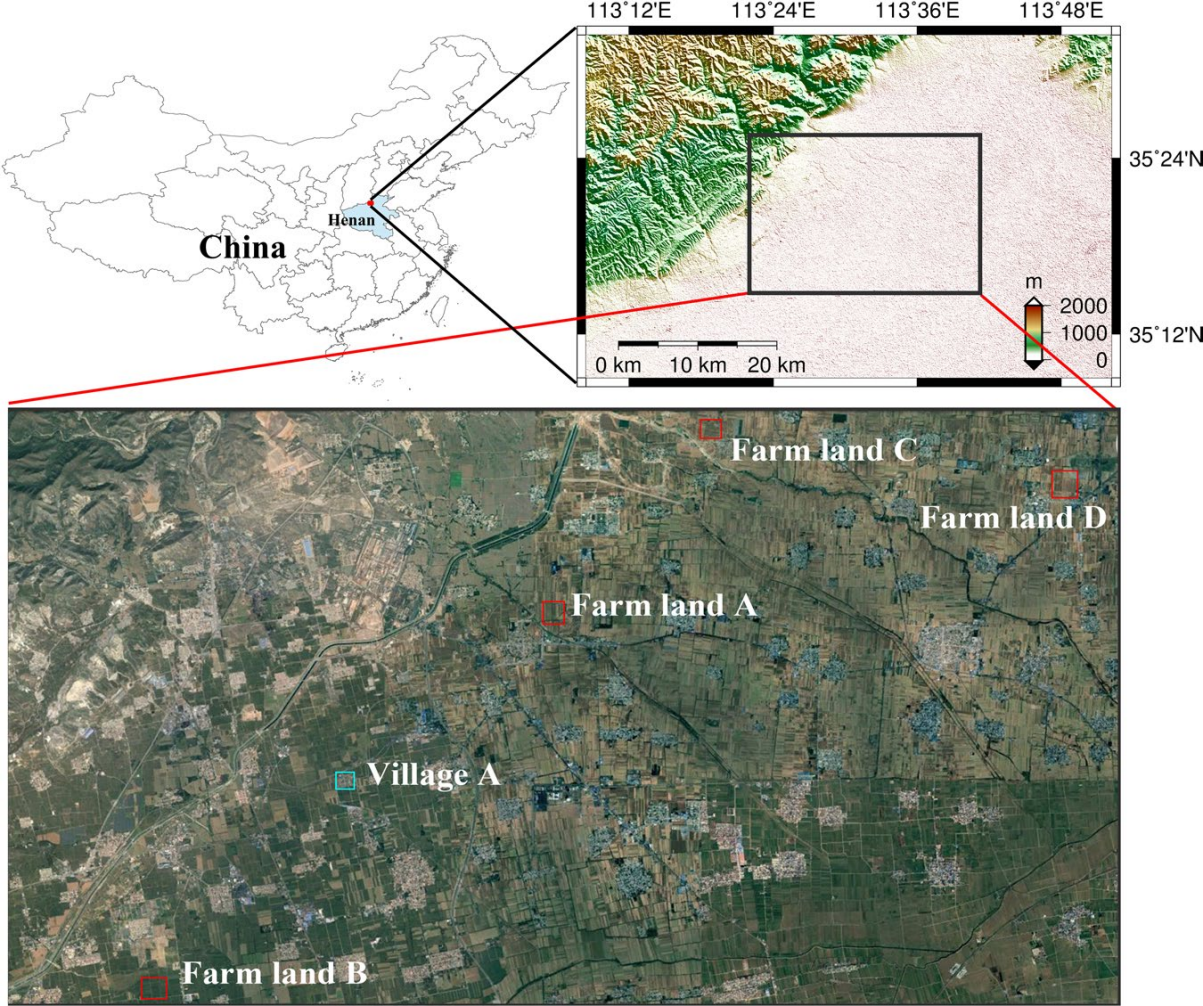
Map of the study area in Henan province. The study area is flat with an average elevation of 90 meters. Optical view of Farm land A, Farm land B, Farm land C, Farm land D and Village A test regions (© Google Earth). Figure done with the ArcMAP 10.3 (https://desktop.arcgis.com/en/arcmap/) and the GMT 5.4 (http://gmt.soest.hawaii.edu/projects/gmt).

**Figure 2:**
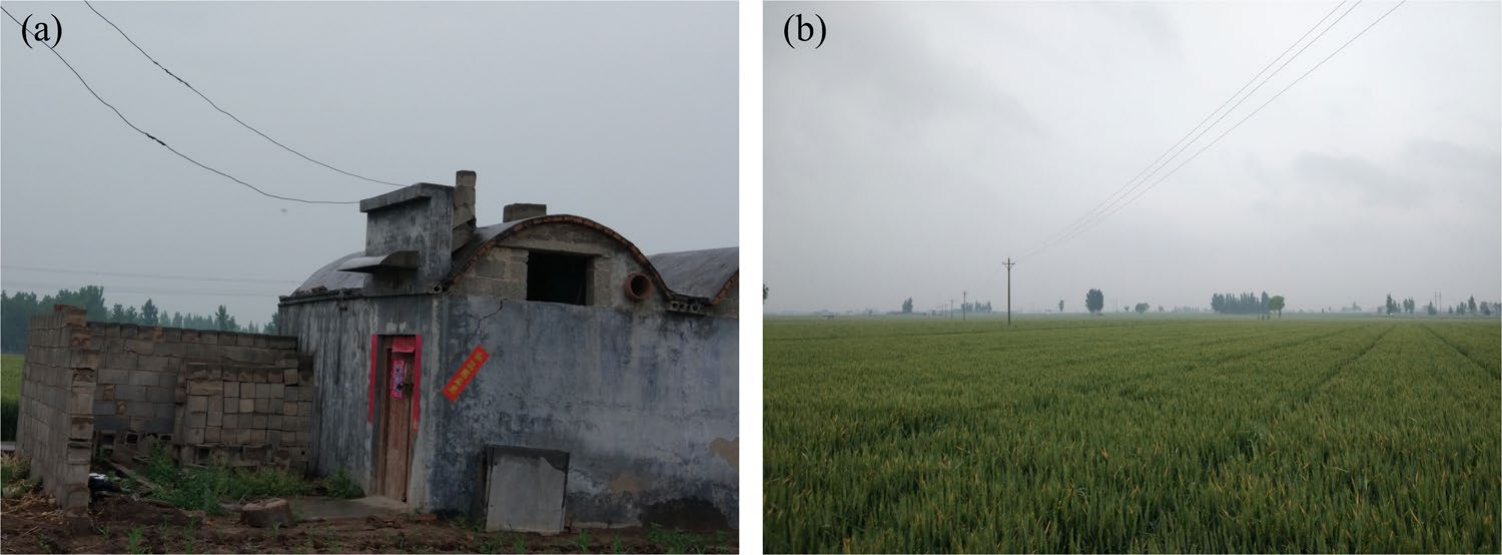
Field photos of the typical ground targets in the spring season. (**a**) The low structure houses are distributed in the village. (**b**) The wheat is planted in spring, while the corn is planted in summer. The picture is the wheat in the spring.

The area of interest in this study is located in Jiaozuo, where is 55 km north of Zhengzhou city (the capital city of Henan) in China (Fig. 1). It belongs to warm temperate zone, semi-humid monsoon climate. The main crops grown in this region are spring wheat and summer corn (Fig. 2). Within the area, four specific areas of farm land and one village are selected for investigation because they have been affected by mine deformation.

### Multi-temporal differential interferograms

DInSAR is based on the interferometric processing of SAR images. The differential interferograms are obtained by the DInSAR method, which used an external digital elevation model (DEM) to remove the topographic effects from the interferogram formed with two SAR images. The interferogram reflects the changes in the geometric properties of ground targets while differential interferogram primarily includes the information pertaining to surface deformation. Combining adjacent SAR images into a consecutive pairs in DInSAR processing is a good way to obtain time series differential interferograms for generation deformation information.

**Figure 3:**
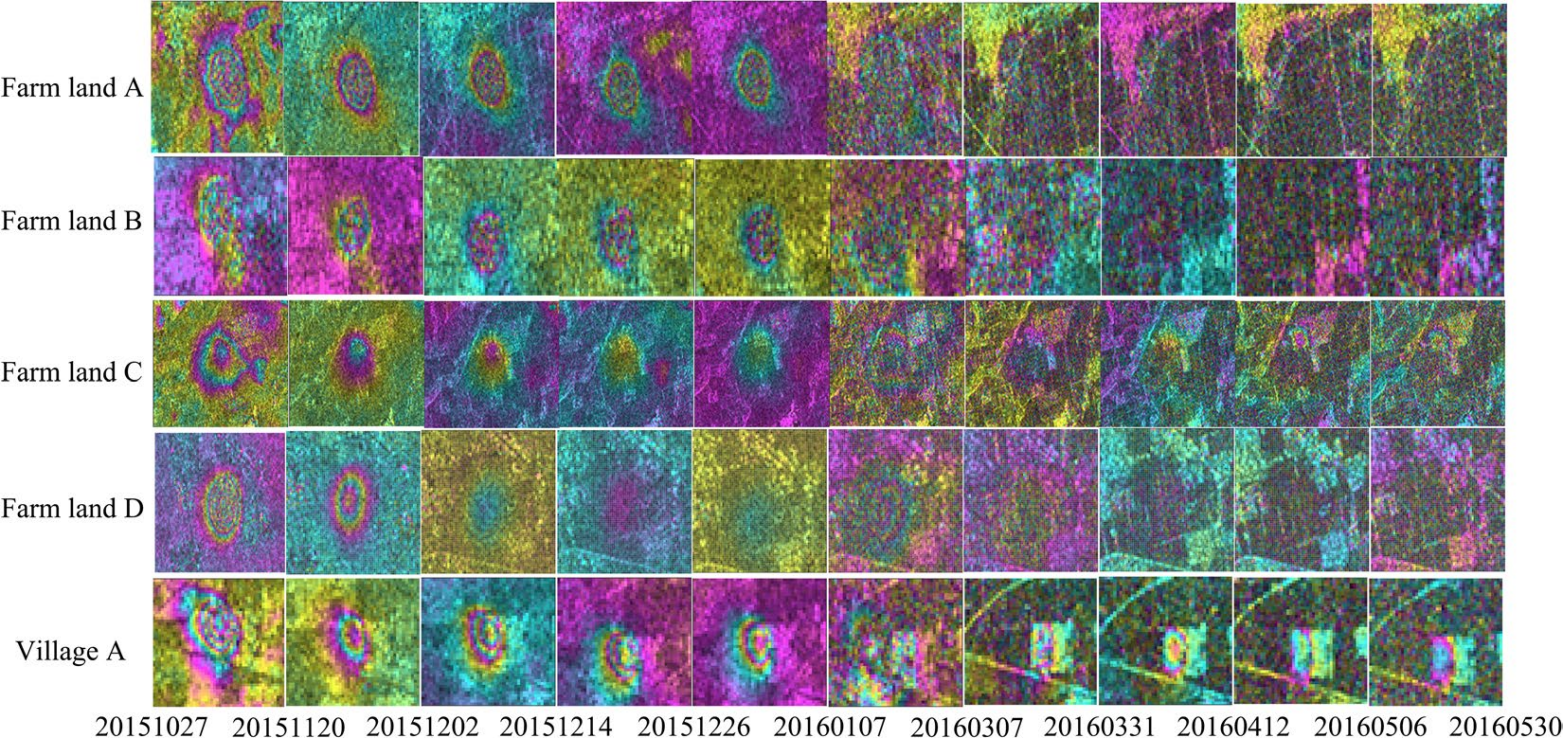
Adjacent images differential interferograms of five study areas in the data set (from October 27, 2015 to May 30, 2016). Horizontal axis is the master image date. Vertical axis is the feature type of the study area. The remaining differential interferograms are in Additional Information Fig. 7.

The 20 differential interferograms of adjacent SAR images along with the corresponding coherence maps were obtained via the DInSAR technique (Figs. 3 and 7). These were derived from a total of 21 images that are available between the months of June 2015 and May 2016. The time between the SAR images range from 12 to 60 d, although the time interval between most of the images is 12 d. It is also important to note the baseline has some influence on the coherence. Due to the high accuracy of the Sentinel-1A satellite orbit, most are within 100 m, although the spatial baseline of one image is as long as 162 m. In this study, the influence of the spatial baseline is ignored. Multi-temporal differential interferograms are a good representation of the deformation in the mining area and the multi-temporal coherence maps provide a good representation of the changes in the surface features.

**Figure 4:**
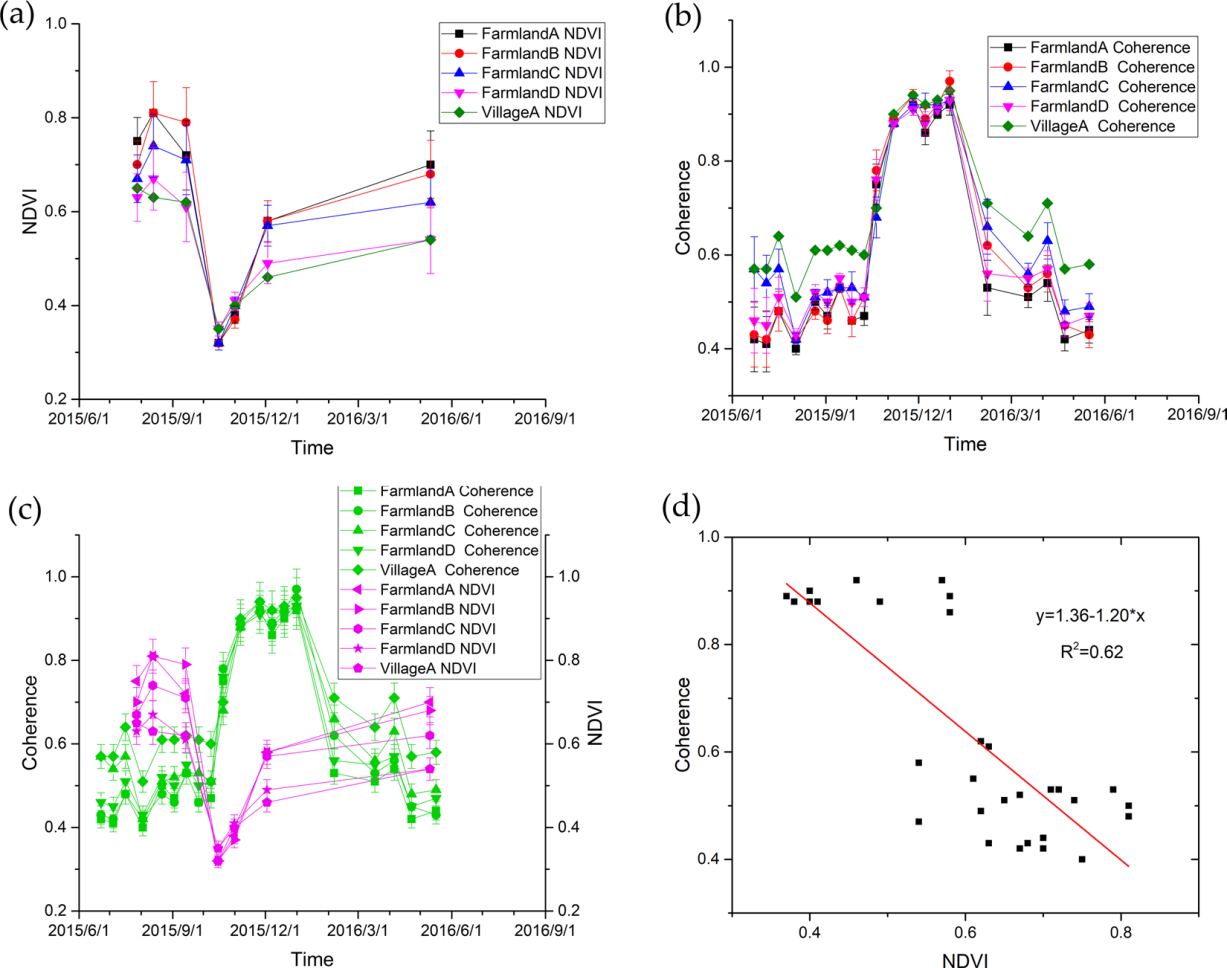
(**a**) Temporal evolution of the NDVI for the farm land and village; (**b**) Temporal evolution of coherence in the farm land and village; (**c**) Comparison of the interferometric coherence and NDVI in farm land and the village; (**d**) Linear fit between the NDVI and coherence. There are thirty NDVI values in total and the data closest to the coherence are selected for comparison.

**Figure 5:**
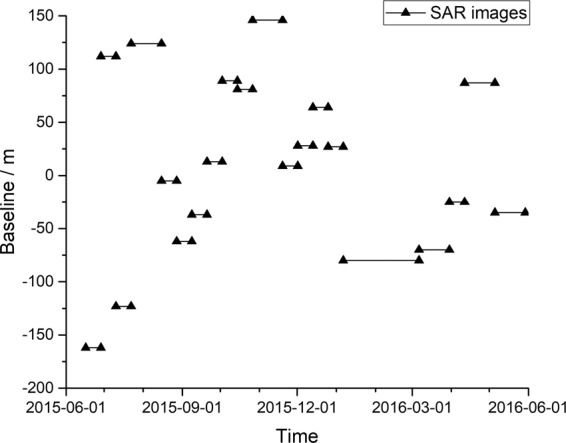
The baseline between 21 Sentinel-1A images. The time interval of the images is mainly 12 days between June 2015 and May 2016. The detailed Sentinel-1A image parameters are shown in Table 2.

**Figure 6:**
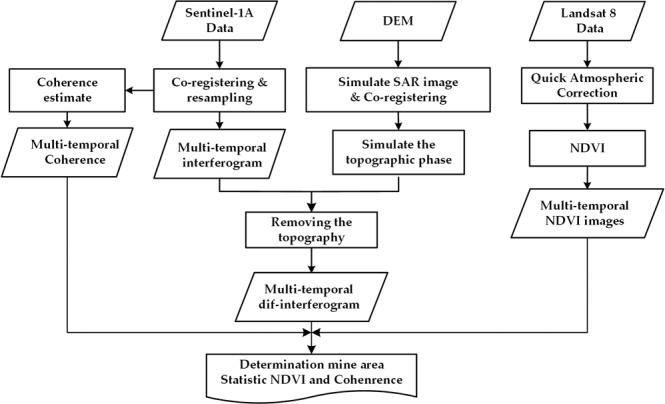
The flow for interferometric coherence and NDVI comparison.

**Figure 7:**
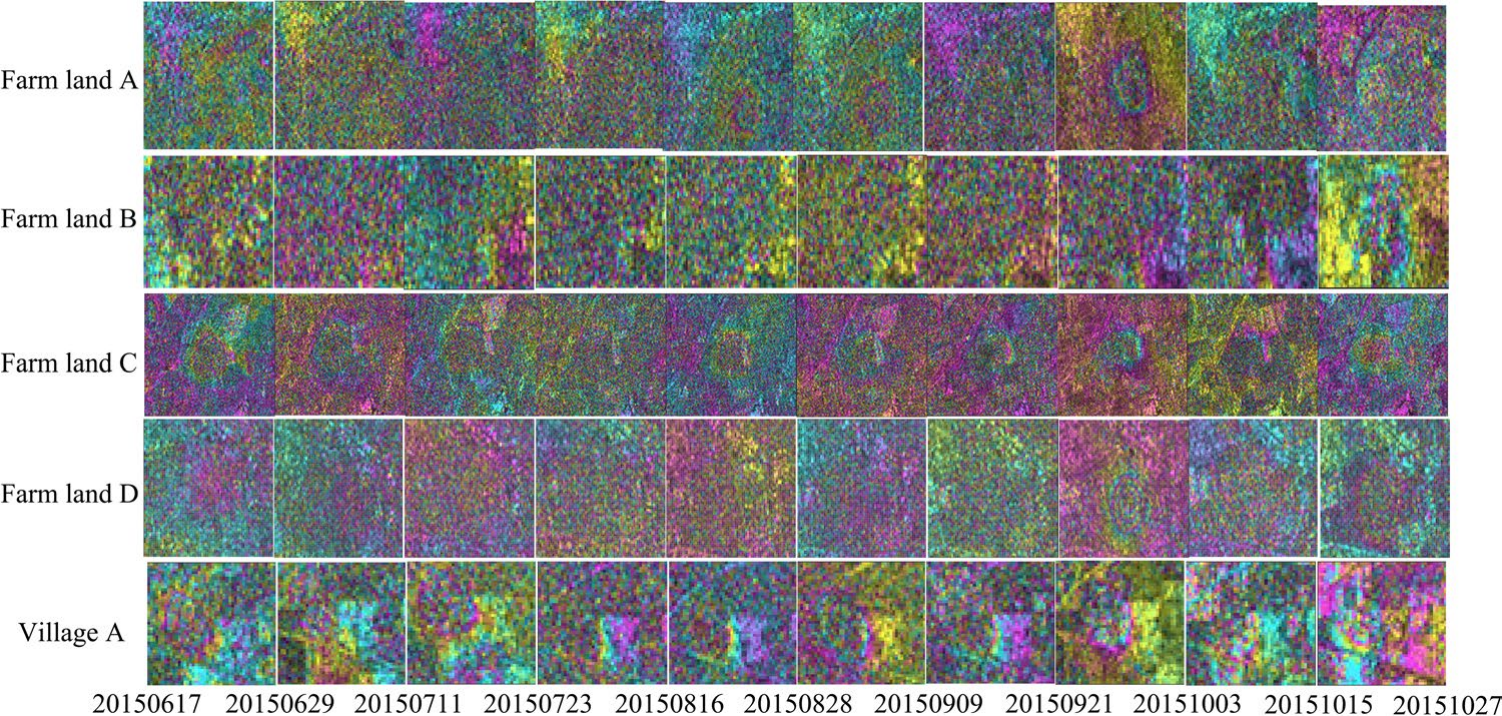
Adjacent images differential interferograms of five study areas in the data set (from June 17, 2015 to October 27, 2015). Horizontal axis is the master image date. Vertical axis is the feature type of the study area.

Figure 3 shows the time series differential interferograms of the farm land and village. A total of 11 groups of interferograms were computed in autumn and winter (August 2015 to January 2016). More obvious deformation fringes appear in the area of Village A from August 2015 to September 2015. The phase fringes are clear and one deformation fringe can be seen (deformation is about 2.8 cm). The differential interferograms can effectively identify the location and extent of deformation. In contrast, the deformation fringes are fuzzy in the farm land areas. The farm land and village have the highest coherence in the winter, which facilitate the accurate calculation of the deformation. There are five sets of interferograms, calculated in the spring time (January 2016 to May 2016). Even with a 60-day interference period (January 2016 to March 2016), good fringe clarity is maintained and up to two deformation fringes can be seen (deformation is about 5.6 cm). The number of fringes in the village area enable effective visual recognition.

### Multi-temporal NDVI values and interferometric coherence

The multi-temporal statistical distribution of the NDVI values in both farm land and village are discussed. Due to the influence of cloud, only 7 images can be used to calculate NDVI. The changes of the NVDI in the farm land and village over one year are shown in Fig. 4a. It has the lowest NDVI values $\approx$ 0.32–0.35 in October. From July to September, the NDVI essentially remains at the same level (NDVI value $\approx$ 0.61–0.81). In general, as shown in this figure, the NVDI values in the village area are obviously lower than those in the farm land.

The 20 coherence of adjacent SAR images were obtained. Figure 4b shows the coherence changes of two surface types in one year. The coherence values are small in summer and spring. As a contrast, it obviously increases in late autumn, while reaches to the highest value (coherence magnitude $\approx$ 0.97 in the farm land and coherence magnitude $\approx$ 0.97 in the village) in winter. A comparison between the two types of land surface shows that the coherence value of the farm land area is markedly lower than that in the village area in the spring and summer. However, the coherence in the autumn and winter remains the same. Therefore these results indicate that the multi-temporal interferometric analysis of the coherence can contribute to the detection of vegetation generally^21^.

We conduct a comparison of the coherence and NDVI values for the five coral subsidence areas derived from the data of Sentinel-1A and Landsat 8. As shown in Fig. 4c, different surface categories have different coherence values and NDVI values. In general, the correlation between the coherence and NDVI is negative for both farm land and the village. In other words, the higher the NDVI value is, the lower the coherence of the interferogram is. The lowest value of NDVI ($\approx$0.32) appears in mid-October, when corns are ripened and leaves wither. Once corns are harvested, the farm land become bare land and the coherence will reach to the highest value (coherence magnitude $\approx$ 0.97) in mid-November. These indicate the NDVI and coherence values have hysteresis in the same area. In December, the NDVI values began to increase when people plant wheat. As the wheat seedlings have little effect on the C-band electromagnetic wave, the SAR images still have high coherence. From summer to autumn (March to September), leaves turn green and crops grow, which induce high NDVI and low coherence in this stage. In August, the NDVI value reaches its maximum point (NDVI value $\approx$ 0.81) while the coherence value reaches its minimum point (coherence magnitude $\approx$ 0.40).

When NDVI and coherence are combined with the differential interferograms shown in Fig. 3, it is found that the NDVI value is high and the coherence value is low in the summer and autumn (i.e. from March to September). During this time, it is not possible to visually determine the location and range of deformation in the farm land areas. In contrast, the location and extent of deformation could be visually identified in the village area, although the deformation fringes are blurred. In winter and spring (October to February), the NDVI value is low and the coherence value is high. In this period, the deformation could be easily visually recognized in both the farm land and village area with clear deformation fringes. As shown in Fig. 4c, both the coherence and NDVI of the farm land and village have seasonal characteristics. The appearance of the farm land change when crops are planted in spring, while the appearance of the village change due to the growth of trees and flowers in the spring and summer.

Upon analysis, a correlation is identified between the interferometric coherence and the NDVI values. We find there is a linear relationships between the interferometric coherence and the NDVI values at the five sites. Figure 4d shows the linear fitting between the NVDI and coherence. A total of thirty NDVI values are selected and those with the closest coherence are used in the comparison. The points represented by the black squares in the figure are used in the linear regression (red line). Since only seven images can be used calculate NDVI values, the correlation coefficient (*R*^2^) between the coherence and NDVI is approximately 0.62, which indicates general agreement between these two distinct measurements.

## Discussion

We focus on what types of deformation can be monitored by DInSAR technology. The coherence is an important concept that provides a good indication of the phase stability of the scatters^5,18^. Within the region of our reseach, the village area exhibits higher coherence than the farm land area (Fig. 4b). In Fig. 3, the differential interferograms have clear phase fringes. The climate in the study area is warm temperate semi-humid monsoon. Vegetation typically grows in spring, becomes luxuriant in summer, declines in autumn, and vanishes in winter. Thus, the coherence of typical ground targets can exhibit obvious seasonal variation, which is consistent with the results of previous studies conducted in other areas^15,22^.

The interferometric coherence maps reflect changes in the physical properties of ground targets. Vegetated areas are usually characterized by volume scattering, which determines low coherence (even for short temporal baseline) due to changes in plant growing stage and movement of stalks and/or leaves caused by wind^14,23^. The NDVI reflects the contrast between different spectral areas. When sunlight strikes vegetation, the chlorophyll in the leaves of the plant strongly absorbs the red portion of the electromagnetic spectrum while the internal structure of the leaves strongly reflects near-infrared light. As shown in Fig. 4c, the NDVI is extremely high due to the rapid growth of crops in the study areas. The interferograms that are only 12 d apart show poor coherence in the summer. Thus, the NDVI values can be reference for elucidating when the traditional DInSAR could be effectively used.

The interferometric coherence and NDVI values are also affected by factors such as crop type, crop phenology status and irrigation levels. In the study, interferometric coherence and NDVI values exhibit an opposite fluctuation in the farm land and the villages (Fig. 4c). Upon analysis, although the mechanisms associated to the calculation of NDVI and coherence are different, they have a negative correlation. Relationships between the interferometric coherence and the NDVI values are observed for the five sites (with coefficient of determination ${R}^{2}$ is 0.62). It indicated that the interferometric coherence can be used as a proxy to fill gaps in NDVI time-series (gaps produced by NDVI unavailability due to cloud cover) for agricultural applications. Coherence can be an important supplementary data, when cloud cover prevents the creation of sufficiently large series NDVI, such as identify a crop type (classification applications), detect agricultural activity (no crop identification needed) or crop yield monitoring.

A total of 24 Landsat 8 images were obtained for this area. However, only 7 of those images which cloud coverage below 11% between the months of June 2015 and June 2016 were used in this study, which was 7 phases of NDVI values in Fig. 4a. These 7 phases NDVI values were not sufficient to cover neither the different values of NDVI nor the four growing season in the whole year, which induced nearly all the NDVI values concentrate on up-left and bottom- right in Fig. 4d. Further works need to use full four season’s optical images to analyze the relationship between the coherence variation and vegetation indexes, as well as on assessing the influence of different bands (X band, C band and L band) and different spatial baseline data on coherence.

## Materials and Methods

### SAR data

The Sentinel-1 constellation provides the European Space Agency (ESA) with complete and updated data in the C band (5.6 cm) for global environmental monitoring purposes^24^. All Sentinel-1 data products are distributed in the single look complex (SLC) format and are available in several acquisition modes, including dual polarization (VV + VH or HH + HV) and single polarization (HH or VV) in strip map (SM) mode and interferometric wide (IW) and extra-wide swath (EW) modes. In the current study, the IW swath mode was used in which the images are recorded in a 250 km $\times$ 250 km area at a 5 m $\times$ 20 m spatial resolution. This mode includes three sub-swaths, namely, IW1, IW2, and IW3, which correspond to cyclical antenna deviations recorded by means of the terrain observation with progressive scans SAR (TOPSAR) technique^25,26^. The high temporal and spatial resolution data provided by Sentinel-1 is well suited to the application described in this study.

The data set consisted of 21 images from ascending orbit 113 (Fig. 5) between June 2015 and May 2016. The data was provided in zero-Doppler slant-range geometry and consist of complex samples that preserved the phase information.

In this study, the interferometric coherence and differential interferograms of the multi-temporal series of Sentinel-1A radar images were obtained by the two-pass DInSAR method^3^, which used an external digital elevation model (DEM) to remove the topographic effects from the interferogram formed with two SAR images. The main steps of the DInSAR technique are depicted in Fig. 6, including SAR image co-registration and resampling, coherence estimation, formation of the interferogram, separation of the deformation information, and determination of the mine area^27–29^. DEM from the Shuttle Radar Topography Mission (SRTM), with 3 arc-sec resolution, was applied for topographic phase removal.

### Optical data

The optical data used in this research was obtained from Landsat 8, which is an American earth observation satellite that was launched on February 11, 2013. The operational land imager (OLI) in Landsat 8 is an improvement on previous generations and provides images spanning nine spectral bands, the resolutions of which are shown in Table 1 to cloud coverage below 11% between the months of June 2015 and June 2016 were used in this study. In addition, Sentinel-2 and China’s GF-1 were also collected. From June 2015 to May 2016, Sentinel-2 and GF-1 had different cloud coverage levels in the study area and could not be used to calculate effective NDVI.

Optical data collected by satellites is influenced by the atmospheric conditions between the satellite and the surface of the Earth, which can degrade the quality of the optical images^30,31^. In order to obtain the actual spectral characteristics of ground features, the ENVI quick atmospheric correction module^32^ was used to remove the effects of the atmosphere in the images.

There are two steps in the processing method adopted in this study (Fig. 6). The first step is to create multi-temporal interferometric coherence maps and differential interferograms. The mining areas were detected via multi-temporal differential interferograms. Subsequently, a statistical comparison of the coherence of the different mining areas was conducted to determine the corresponding correlation. The second step is to calculate the NDVI in the area corresponding to the location of the mine identified via the DInSAR method. A statistical comparison of the multi-temporal NDVI was conducted to detect the areas of plant growth. A correlation analysis was performed between the multi-temporal interferometric coherence and the NDVI with regard to the temporal variability of the vegetation. In addition, in order to confirm the accuracy of the coherence and NDVI, the standard deviations of the coherence values and the NDVI values were calculated for the five areas containing farm land.

### Vegetation indices

The vegetation indices obtained from the optical images provide important information regarding its structure and composition^33,34^. It is well known that when sunlight strikes vegetation, the chlorophyll in the leaves of the plant strongly absorbs the red portion of the electromagnetic spectrum (from 0.4 to 0.7 *μ*m) while the internal structure of the leaves strongly reflects near-infrared light (from 0.7 to 1.1 *μ*m)^35^. The contrast between the different spectral areas can be used to estimate the greenness of the vegetation as follows:

$$NDVI=\frac{NIR-RED}{NIR+RED}$$

The NDVI is a continuum of pixel values ranging from minus one (−1) to plus one (+1). In general, negative values indicate no vegetation while positive values indicate the presence of vegetation. Values close to +1 indicate the highest possible density of green leaves.

**Table 1:** Landset 8 images data set-font bold and marked * images were used in this study.

Image Number	Acquisition Date	Cloudiness (%)	Path	Spatial resolution
1	10 June 2015	23.52	124	30 m
2	26 June 2015	72.36	124	30 m
3	12 July 2015	23.33	124	30 m
4*	28 July 2015	6.16	124	30 m
5*	13 August 2015	10.45	124	30 m
6	29 August 2015	13.79	124	30 m
7*	14 September 2015	0.18	124	30 m
8	30 September 2015	76.15	124	30 m
9*	16 October 2015	8.09	124	30 m
10*	1 November 2015	0.13	124	30 m
11	17 November 2015	100	124	30 m
12*	3 December 2015	0.13	124	30 m
13	19 December 2015	99.97	124	30 m
14	4 January 2016	97.92	124	30 m
15	20 January 2016	98.60	124	30 m
16	5 February 2016	45.03	124	30 m
17	21 February 2016	98.33	124	30 m
18	8 March 2016	85.74	124	30 m
19	24 March 2016	34.25	124	30 m
20	9 April 2016	75.17	124	30 m
21	25 April 2016	89.43	124	30 m
22*	11 May 2016	3.06	124	30 m
23	27 May 2016	39.56	124	30 m
24	12 June 2016	86.83	124	30 m

**Table 2:** Sentinel-1A image parameters.

Image Number	Acquisition Date	Spatial Baseline (m)	Temporal Baseline (Day)
1	17 June 2015	0	0
2	29 June 2015	−162	12
3	11 July 2015	112	12
4	23 July 2015	−123	12
5	16 August 2015	124	24
6	28 August 2015	−5	12
7	9 September 2015	−62	12
8	21 September 2015	−37	12
9	3 October 2015	13	12
10	15 October 2015	89	12
11	27 October 2015	81	12
12	20 November 2015	146	24
13	2 December 2015	9	12
14	December 2015	28	12
15	26 December 2015	64	12
16	7 January 2016	27	12
17	7 March 2016	−80	60
18	31 March 2016	−70	24
19	12 April 2016	−25	12
20	6 May 2016	87	24
21	30 May 2016	−35	24

### Multi-temporal SAR images

In interferometry, an interferogram can be obtained by multiplying the complex information in one image by the complex conjugate information of a second image to form an interferogram, and a differential interferogram can be obtained by removing the reference DEM and replacing it with precise orbital data^36^.

In terms of how they are used, interferograms and coherence maps respectively reflect changes in the geometric and physical properties of ground targets while differential interferograms primarily include information pertaining to surface deformation. Note that a pair of images is required when the objective is to monitor deformation. However, the loss of InSAR coherence prevents the measurement of ground surface deformation. Temporal decorrelation is a major factor affecting coherence, although this can be mitigated by choosing an image pair with a short baseline and a test area with minimal vegetation^37,38^.

The interferometric coherence is a cross-correlation product calculated from two co-registered complex SAR images over a small window of pixels^39,40^, which can be mathematically represented as follows:

$$\gamma =\frac{E({s}_{1}{s}_{2}^{\ast })}{\sqrt{E({s}_{1}{s}_{1}^{\ast })E({s}_{2}{s}_{2}^{\ast })}}$$

where *s*_1_ and *s*_2_ are pixel values, the superscript * denotes the conjugate complex, and *E*(•) represents the expected value.
